# A duality principle for the multi-block entanglement entropy of free fermion systems

**DOI:** 10.1038/s41598-017-09550-1

**Published:** 2017-09-11

**Authors:** J. A. Carrasco, F. Finkel, A. González-López, P. Tempesta

**Affiliations:** 1 Departamento de Física Teórica II, Universidad Complutense de Madrid, 28040 Madrid, Spain; 2 Instituto de Ciencias Matemáticas (CSIC–UAM–UC3M–UCM), c/Nicolás Cabrera 13–15, 28049 Madrid, Spain

## Abstract

The analysis of the entanglement entropy of a subsystem of a one-dimensional quantum system is a powerful tool for unravelling its critical nature. For instance, the scaling behaviour of the entanglement entropy determines the central charge of the associated Virasoro algebra. For a free fermion system, the entanglement entropy depends essentially on two sets, namely the set *A* of sites of the subsystem considered and the set *K* of excited momentum modes. In this work we make use of a general duality principle establishing the invariance of the entanglement entropy under exchange of the sets *A* and *K* to tackle complex problems by studying their dual counterparts. The duality principle is also a key ingredient in the formulation of a novel conjecture for the asymptotic behavior of the entanglement entropy of a free fermion system in the general case in which both sets *A* and *K* consist of an arbitrary number of blocks. We have verified that this conjecture reproduces the numerical results with excellent precision for all the configurations analyzed. We have also applied the conjecture to deduce several asymptotic formulas for the mutual and *r*-partite information generalizing the known ones for the single block case.

## Introduction

One of the distinguishing features of the quantum realm is the existence of entangled states in composite systems, which have no classical analogue and play a fundamental role in quantum information theory and condensed matter physics (see, e.g., refs 1, 2). A widely used quantitative measure of the degree of entanglement between two subsystems *A*, *B* of a quantum system *A* ∪ *B* in a pure state *ρ* = |*ψ*〉〈*ψ*| is the Rényi entanglement entropy^3^ ${S}_{\alpha }(A)={\mathrm{(1}-\alpha )}^{-1}\,\mathrm{log}\,{\rm{tr}}({\rho }_{A}^{\alpha })$, where *ρ*_*A*_ is the reduced density matrix of the subsystem *A* and *α* > 0 is the Rényi parameter (the von Neumann entropy is obtained in the limit *α* → 1). It is easy to show that *S*_*α*_(*A*) = *S*_*α*_(*B*), and that the entanglement entropy vanishes when the whole system is in a non-entangled (product) state. Over the last decade, it has become clear that the study of the entanglement between two extended subsystems of a many-body system in one dimension is a powerful tool for uncovering its criticality properties^4–7^. The reason for this is that one-dimensional critical quantum systems are governed by an effective conformal field theory (CFT) in (1 + 1) dimensions, whose entanglement entropy can be evaluated in closed form in the thermodynamic limit^8–10^. In the simplest case, when the subsystem *A* consists of a single interval of length *L* and the whole system is in its ground state, the scaling of *S*_*α*_(*A*) for *L* → ∞ is determined solely by the central charge *c*. In order to probe the full operator content of the CFT, one needs to analyze more complicated situations in which the set *A* is the union of a finite number of intervals. In fact, in the last few years there has been a considerable interest in this problem, both for CFTs and one-dimensional lattice models (integrable spin chains or free fermion systems), as witnessed by the number of papers published on this subject (see, e.g., refs 11–18).

In this work we shall extend this analysis to the more general case in which the system’s state is also made up of several blocks of consecutive excited momentum modes, which has received comparatively less attention^19–24^. An important motivation for dealing with this type of states is that it makes it possible to treat position and momentum space on a more equal footing, thus revealing certain symmetries that have not been fully exploited so far. This approach naturally leads to a duality principle for the behavior of the entanglement entropy under the exchange of the position and momentum space block configurations, which in fact can be exploited to solve problems that up until now had defied an analytic treatment^25^ with standard techniques like the Fisher–Hartwig conjecture^26^. We have applied this duality principle to propose a new conjecture on the composability of the entanglement entropy in the multi-block case, which yields a closed asymptotic formula for the Rényi entanglement entropy of a free fermion system in the most general multi-block configuration, both in position and momentum space. This formula, which we have numerically verified for a wide range of configurations both for 0 < *α* < 1 and $\alpha \ge 1$, reduces to the known ones when the configuration in momentum space consists of a single block. It also leads to closed asymptotic formulas for the mutual and the tripartite^12^ (or *r*-partite^18^) information, which again agree with the general CFT predictions.

## Results and Methods

### Preliminaries and notation

The model considered is a system of *N* free (spinless) hopping fermions with creation operators ${a}_{j}^{\dagger }$ (where the subindex *j* = 0, …, *N* − 1 denotes the site) and Hamiltonian $H={\sum }_{i,j=0}^{N-1}{g}_{N}(i-j){a}_{i}^{\dagger }{a}_{j}$ preserving the total fermion number. We shall further assume that the hopping amplitude *g*_*N*_ satisfies *g*_*N*_(*k*) = *g*_*N*_(−*k*)^*^ = *g*_*N*_(*k* + *N*), so that *H* is Hermitian and translationally invariant. For this reason, it is convenient to introduce the Fourier-transformed creation operators

1 $${\hat{a}}_{j}^{\,\dagger }=\frac{1}{\sqrt{N}}\sum _{l=0}^{N-1}{{\rm{e}}}^{2\pi {\rm{i}}jl/N}{a}_{l}^{\dagger },\quad 0\le j\le N-1.$$

It is straightforward to check that the operators ${\widehat{a}}_{j}$, ${\widehat{a}}_{j}^{\,\dagger }$ satisfy the canonical anticommutation relations (CAR), and that they diagonalize *H*. In fact, we have $H={\sum }_{l=0}^{N-1}{\varepsilon }_{N}(l){\widehat{a}}_{l}^{\,\dagger }{\widehat{a}}_{l}\,,$ with ${\varepsilon }_{N}(l)={\sum }_{j\mathrm{=0}}^{N-1}{g}_{N}(j){{\rm{e}}}^{2\pi {\rm{i}}jl/N}\,\mathrm{.}$ It can be shown that the total momentum operator *P* is also diagonal in this representation, namely $P={\sum }_{l=0}^{N-1}{p}_{l}{\widehat{a}}_{l}^{\,\dagger }{\widehat{a}}_{l}$, with *p*_*l*_ = 2*πl*/*N* mod 2*π*. Thus the operator ${\widehat{a}}_{l}^{\,\dagger }$ creates a (non-localized) fermion with well-defined energy *ε*_*N*_(*l*) and momentum *p*_*l*_. Note that *ε*_*N*_(*l*) is obviously real for all modes *l*, and that the model is critical (gapless) if *ε*_*N*_(*l*) vanishes for some *l*. We shall suppose in what follows that the system is in a pure energy eigenstate

2 $$|K\rangle \equiv {\widehat{a}}_{{k}_{1}}^{\,\dagger }\cdots {\widehat{a}}_{{k}_{M}}^{\,\dagger }|0\rangle ,\quad K=\{{k}_{1},\ldots ,{k}_{M}\}\subset \{0,\ldots ,N-1\},$$

where |0〉 is the vacuum, consisting of *M* fermions with momenta 2*πk*_*j*_/*N*. We shall be interested in studying the entanglement properties of a subset of sites *A* ≡ {*x*_1_, …, *x*_*L*_} ⊂ {0, …, *N* − 1} with respect to the whole system when the latter is in the pure state |*K*〉. As is well known, these properties are encoded in the reduced density matrix *ρ*_*A*_ = tr_*B*_ *ρ*, where *ρ* ≡ |*K*〉〈*K*| and *B* = {0, …, *N* − 1} − *A*. As mentioned in the Introduction, the degree of entanglement is usually measured using the Rényi entanglement entropy ${S}_{\alpha }(A)\equiv {\mathrm{(1}-\alpha )}^{-1}\,\mathrm{log}\,{\rm{tr}}({\rho }_{A}^{\alpha })$ (with *α* > 0). One of the most efficient ways of computing this entropy is to exploit the connection between the reduced density matrix *ρ*_*A*_ and the correlation matrix *C*_*A*_, defined by

3 $${({C}_{A})}_{jk}=\langle K|{a}_{{x}_{j}}^{\dagger }{a}_{{x}_{k}}|K\rangle ,\quad 1\le j,k\le L\,\mathrm{.}$$

This matrix is obviously Hermitian, with eigenvalues *ν*_1_, …, *ν*_*L*_ lying in the interval [0, 1]. Moreover, since the state |*K*〉 is determined by the conditions ${\widehat{a}}_{k}^{\,\dagger }|K\rangle =0$ for *k* ∈ *K* and ${\widehat{a}}_{k}|K\rangle =0$ for $k\notin K$, the expectation value $\langle K|{\widehat{a}}_{j}^{\,\dagger }{\widehat{a}}_{k}|K\rangle$ vanishes for *k* ∉ *K* and equals *δ*_*jk*_ for *k* ∈ *K*. From this fact and Eq. (1) we immediately obtain the following explicit expression for the matrix elements of the correlation matrix *C*_*A*_:

4 $${({C}_{A})}_{jk}=\frac{1}{N}\sum _{l\in K}{{\rm{e}}}^{-2\pi {\rm{i}}({x}_{j}-{x}_{k})l/N},\quad 1\le j,k\le L\,\mathrm{.}$$

As first shown in refs 4, 27, the reduced density matrix *ρ*_*A*_ factors as the tensor product ${\rho }_{A}={\otimes }_{l=1}^{L}{\rho }_{A}^{(l)}$, where each ${\rho }_{A}^{(l)}$ is a 2 × 2 density matrix with eigenvalues *ν*_*l*_ and 1 − *ν*_*l*_. In particular, the spectrum of *ρ*_*A*_ is the set of numbers

5 $${\rho }_{A}({\varepsilon }_{1},\ldots ,{\varepsilon }_{L})=\prod _{l=1}^{L}[{\nu }_{l}^{\,{\varepsilon }_{l}}{\mathrm{(1}-{\nu }_{l})}^{1-{\varepsilon }_{l}}]\,,\quad {\varepsilon }_{l}\in \mathrm{\{0,\; 1\}.}$$

Since the Rényi entropy *S*_*α*_ is additive, it follows that

6 $${S}_{\alpha }(A)=\sum _{l=1}^{L}{S}_{\alpha }({\rho }_{A}^{(l)})={(1-\alpha )}^{-1}\sum _{l=1}^{L}\,{\rm{l}}{\rm{o}}{\rm{g}}({\nu }_{l}^{\alpha }+{(1-{\nu }_{l})}^{\alpha }).$$

Note that the latter method for computing *S*_*α*_(*A*) is computationally very advantageous, since it is based on the diagonalization of the *L* × *L* matrix *C*_*A*_ as opposed to direct diagonalization of the 2^*L*^ × 2^*L*^ matrix *ρ*_*A*_.

As explained above, it is of great interest to determine the (leading) asymptotic behaviour of the entanglement entropy *S*_*α*_(*A*) as the size *L* of the subsystem *A* tends to infinity. To this end, note first of all that the matrix *C*_*A*_ is Toeplitz (i.e., (*C*_*A*_)_*jk*_ depends only on the difference *j* − *k*) provided that the subsystem *A* under consideration is a single block, i.e., a set of consecutive sites. Let us further assume that Eq. (4) has a well-defined limit as *N* → ∞ with *L* fixed, in the sense that there exists a piecewise smooth density function *c*(*p*) such that ${({C}_{A})}_{jk}\to {\mathrm{(2}\pi )}^{-1}{\int }_{0}^{2\pi }c(p)\,{{\rm{e}}}^{-{\rm{i}}(j-k)p}{\rm{d}}p$ in this limit. As first shown by Jin and Korepin^5^, it is then possible to apply a particular case of the Fisher–Hartwig conjecture^26^ proved by Basor^28^ to derive an asymptotic formula for the characteristic polynomial of the correlation matrix *C*_*A*_, and hence for the entanglement entropy *S*_*α*_(*A*) (see also refs 23, 24, 29). However, when the subsystem *A* is not a single block it is clear from Eq. (4) that *C*_*A*_ is not a Toeplitz matrix, and therefore the method just outlined cannot be used to derive the asymptotic behaviour of *S*_*α*_(*A*) for large *L*. It should also be stressed that the asymptotic result in ref. 5 is only valid for $N\gg L\gg 1$ (i.e., for an *infinite* chain), since the *N* → ∞ limit with *L* fixed is taken before letting *L* → ∞. In particular, the asymptotic behaviour of *S*_*α*_(*A*) when *N* → ∞ with *L*/*N* → *γ*_*x*_ ∈ (0, 1) cannot be directly inferred from the latter result. As we shall explain shortly, these drawbacks can be overcome through the use of a duality principle that we shall introduce below.

### The dual correlation matrix

We start by defining the projection of the operator ${\widehat{a}}_{j}^{\,\dagger }$ onto the set ${\mathcal L} ({ {\mathcal H} }_{A})$ of linear operators from the Hilbert space ${ {\mathcal H} }_{A}$ of the subsystem *A* into itself in the obvious way, namely (cf. Eq. (1))

7 $${\widehat{a}}_{A,j}^{\dagger }=\frac{1}{\sqrt{N}}\sum _{l\in A}{{\rm{e}}}^{2\pi {\rm{i}}jl/N}{a}_{l}^{\dagger },$$

and similarly for ${\widehat{a}}_{A,j}$. We shall also denote by ${\widehat{a}}_{B,j}^{\,\dagger }$, ${\widehat{a}}_{B,j}$ the corresponding projections onto ${\mathcal L} ({ {\mathcal H} }_{B})$, so that ${\widehat{a}}_{j}={\widehat{a}}_{A,j}+{\widehat{a}}_{B,j},$ ${\widehat{a}}_{j}^{\,\dagger }={\widehat{a}}_{A,j}^{\,\dagger }+{\widehat{a}}_{B,j}^{\,\dagger }$. We then define the *dual correlation matrix* ${\widehat{C}}_{A}$ as the *M* × *M* matrix with elements

8 $${({\widehat{C}}_{A})}_{lm}=\langle 0|{\widehat{a}}_{A,{k}_{l}}{\widehat{a}}_{A,{k}_{m}}^{\dagger }|0\rangle ,\quad 1\le l,m\le M\mathrm{.}$$

The dual correlation matrix ${\widehat{C}}_{B}$ of the complementary set *B* is defined similarly. The analogue of the matrix ${\widehat{C}}_{A}$ for continuous systems, usually called the overlap matrix, was originally introduced by Klich^30^ and has been extensively used in the literature (see, e.g., ref. 31). From the definition (7) of the projected operators ${\widehat{a}}_{A,j}^{\dagger }$ we immediately obtain the explicit formula

9 $${({\widehat{C}}_{A})}_{lm}=\frac{1}{N}\sum _{j\in A}{{\rm{e}}}^{-2\pi {\rm{i}}({k}_{l}-{k}_{m})j/N},\quad 1\le l,m\le M\mathrm{.}$$

Comparison with Eq. (4) shows that ${\widehat{C}}_{A}$ is obtained from *C*_*A*_ by exchanging the roles played by the sites *x*_*j*_ ∈ *A* and the excited modes *k*_*l*_ ∈ *K*, which justifies the term “dual correlation matrix”. We shall show in what follows that this duality can be successfully exploited to obtain the asymptotic behaviour of *S*_*α*_(*A*) in situations in which the usual approach based on the correlation matrix *C*_*A*_ is not feasible.

The matrix ${\widehat{C}}_{A}$ is clearly Hermitian and positive semidefinite, since for all ${z}_{1},\ldots ,{z}_{M}\in {\mathbb{C}}$ we have ${\sum }_{l,m=1}^{M}{({\hat{C}}_{A})}_{lm}\,$ ${z}_{l}^{\ast }{z}_{m}={\parallel ({\sum }_{l=1}^{M}{z}_{m}{\hat{a}}_{A,{k}_{m}}^{\dagger })|0\rangle \parallel }^{2}\,$. Thus the eigenvalues ${\widehat{\nu }}_{1},\ldots ,{\widehat{\nu }}_{M}$ of ${\widehat{C}}_{A}$ are nonnegative. Using the identities $\langle 0|{{\mathscr{O}}}_{A}{{\mathscr{O}}}_{B}^{^{\prime} }|0\rangle =\langle 0|{{\mathscr{O}}}_{B}^{^{\prime} }{{\mathscr{O}}}_{A}|0\rangle =\mathrm{0,}$ where ${{\mathscr{O}}}_{A}$ and ${{\mathscr{O}}}_{B}^{^{\prime} }$ are linear operators respectively supported on *A* and *B*, it is straightforward to check that ${\hat{C}}_{B}={{\mathbb{1}}}_{M}-{\hat{C}}_{A}.$ Since ${\widehat{C}}_{B}$ is also positive semidefinite, from the previous relation it follows that ${\widehat{\nu }}_{i}\in \mathrm{[0},\mathrm{1]}$ for all *i* = 1, …, *M*. Moreover, the Hermitian character of ${\widehat{C}}_{A}$ implies that there exists a unitary *M* × *M* matrix $U\equiv {({u}_{lm})}_{1\le l,m\le M}$ such that $U{\widehat{C}}_{A}{U}^{\dagger }={\rm{diag}}({\widehat{\nu }}_{1},\ldots ,{\widehat{\nu }}_{M})$, and hence $U{\hat{C}}_{B}{U}^{\dagger }={\mathbb{1}}-U{\hat{C}}_{A}{U}^{\dagger }={\rm{d}}{\rm{i}}{\rm{a}}{\rm{g}}(1-{\hat{\nu }}_{1},\ldots ,1-{\hat{\nu }}_{M})$. We then define the corresponding rotated operators ${\widehat{c}}_{l}={\sum }_{m=1}^{M}{u}_{lm}\,{\widehat{a}}_{{k}_{m}}$ ($1\le l\le M$), which together with their adjoints satisfy the CAR by the unitarity of *U*. We shall also need the projections of the latter operators onto the spaces ${\mathcal L} ({ {\mathcal H} }_{A})$ and ${\mathcal L} ({ {\mathcal H} }_{B})$, namely

10 $${\widehat{c}}_{A,l}=\sum _{m=1}^{M}{u}_{lm}{\widehat{a}}_{A,{k}_{m}},\quad {\widehat{c}}_{B,l}=\sum _{m=1}^{M}{u}_{lm}\,{\widehat{a}}_{B,{k}_{m}}={\widehat{c}}_{l}-{\widehat{c}}_{A,l},$$

and similarly for their adjoints. From the above definitions it follows that the vacuum correlators of the operators $\{{\widehat{c}}_{A,l},{\widehat{c}}_{A,l}^{\,\dagger }\}$ and $\{{\widehat{c}}_{B,l},{\widehat{c}}_{B,l}^{\,\dagger }\}$ are given by

11 $$\langle 0|{\widehat{c}}_{A,l}{\widehat{c}}_{A,m}^{\,\dagger }|0\rangle ={\widehat{\nu }}_{l}{\delta }_{lm},\quad \langle 0|{\widehat{c}}_{B,l}{\widehat{c}}_{B,m}^{\,\dagger }|0\rangle =\mathrm{(1}-{\widehat{\nu }}_{l}){\delta }_{lm},$$

and hence ${\Vert {\widehat{c}}_{A,l}^{\dagger }|0\rangle \Vert }^{2}={\widehat{\nu }}_{l}$, ${\Vert {\widehat{c}}_{B,l}^{\dagger }|0\rangle \Vert }^{2}=1-{\widehat{\nu }}_{l}$. Following ref. 30, we note that the state $|\varphi \rangle ={\widehat{c}}_{1}^{\,\dagger }\cdots {\widehat{c}}_{M}^{\,\dagger }|0\rangle$ actually differs from |*K*〉 by an irrelevant phase, since by definition of the operators ${\widehat{c}}_{l}$ we have

$$|\varphi \rangle =\sum _{{m}_{1},\ldots ,{m}_{M}=1}^{M}{u}_{1{m}_{1}}^{\ast }\cdots {u}_{M,{m}_{M}}^{\ast }{\hat{a}}_{{k}_{{m}_{1}}}^{\,\dagger }\cdots {\hat{a}}_{{k}_{{m}_{M}}}^{\,\dagger }|0\rangle =(\sum _{\sigma \in {S}_{M}}{(-1)}^{\sigma }{u}_{1{\sigma }_{1}}^{\ast }\cdots {u}_{M,{\sigma }_{M}}^{\ast }){\hat{a}}_{{k}_{1}}^{\,\dagger }\cdots {\hat{a}}_{{k}_{M}}^{\,\dagger }|0\rangle =det{U}^{\ast }|K\rangle ,$$

where (−1)^*σ*^ denotes the sign of the permutation *σ*. The latter relation implies that |*K*〉〈*K*| = |*ϕ*〉〈*ϕ*|, a fact that can be exploited in order to derive an expression for the entanglement entropy *S*_*α*_(*A*). To this end, for ${\widehat{\nu }}_{l}\ne 0,1$ we define the operators ${\widehat{d}}_{A,l}^{\dagger }={\widehat{c}}_{A,l}^{\,\dagger }/\sqrt{{\widehat{\nu }}_{l}}$, ${\widehat{d}}_{B,l}^{\dagger }={\widehat{c}}_{B,l}^{\dagger }/\sqrt{1-{\widehat{\nu }}_{l}}$, so that by Eq. (11) the states ${|1\rangle }_{A,l}\equiv {\widehat{d}}_{A,l}^{\dagger }|0\rangle$, ${|1\rangle }_{B,l}\equiv {\widehat{d}}_{B,l}^{\dagger }|0\rangle$ are properly normalized. On the other hand, when ${\widehat{\nu }}_{l}=0$ the state ${\widehat{c}}_{l}^{\,\dagger }|0\rangle ={\widehat{c}}_{B,l}^{\,\dagger }|0\rangle$ is supported on *B* by Eq. (11), and is normalized, since the operators ${\widehat{c}}_{l},{\widehat{c}}_{l}^{\,\dagger }$ obey the CAR. Hence in this case we simply set ${\widehat{d}}_{B,l}^{\dagger }={\widehat{c}}_{B,l}^{\,\dagger }={\widehat{c}}_{l}^{\,\dagger }$, ${|1\rangle }_{B,l}={\widehat{d}}_{B,l}^{\dagger }|0\rangle$. Similarly, when ${\widehat{\nu }}_{l}=1$ we define ${\widehat{d}}_{A,l}^{\dagger }={\widehat{c}}_{A,l}^{\,\dagger }={\widehat{c}}_{l}^{\,\dagger }$, ${|1\rangle }_{A,l}={\widehat{d}}_{A,l}^{\dagger }|0\rangle$, and by the previous definitions we thus have ${\widehat{c}}_{l}^{\,\dagger }=\sqrt{{\widehat{\nu }}_{l}}{\widehat{d}}_{A,l}^{\dagger }+\sqrt{1-{\widehat{\nu }}_{l}}{\widehat{d}}_{B,l}^{\dagger }$ ($1\le l\le M$), and therefore $|\varphi \rangle ={\otimes }_{l=1}^{M}(\sqrt{{\hat{\nu }}_{l}}{|1\rangle }_{A,l}{|0\rangle }_{B,l}+\sqrt{1-{\hat{\nu }}_{l}}$ ${|0\rangle }_{A,l}{|1\rangle }_{B,l})$,where |0〉_*A*,*l*_, |0〉_*B*,*l*_ denote the vacuum state in the *l*-th mode (with respect to the ${\widehat{c}}_{m}^{\,\dagger }$ operators) supported respectively on *A* or *B*. Using the identity |*K*〉〈*K*| = |*ϕ*〉〈*ϕ*| and tracing over the degrees of freedom of the subsystem *B* we easily arrive at the fundamental formula

12 $${\rho }_{A}=\underset{l=1}{\overset{M}{\otimes }}({\widehat{\nu }}_{l}{|1\rangle }_{A,l}{\langle 1|}_{A,l}+(1-{\widehat{\nu }}_{l}){|0\rangle }_{A,l}{\langle 0|}_{A,l})\,.$$

In particular, the spectrum of the matrix *ρ*_*A*_ is the set of numbers

13 $${\rho }_{A}({\varepsilon }_{1},\ldots ,{\varepsilon }_{M})=\prod _{l=1}^{M}[{\hat{\nu }}_{l}^{\,{\varepsilon }_{l}}{(1-{\hat{\nu }}_{l})}^{1-{\varepsilon }_{l}}]\,,\quad {\varepsilon }_{l}\in \{0,1\}\,,$$

up to zero eigenvalues. From the additivity of the Rényi entropy and Eqs (12) or (13) it follows that the entanglement entropy *S*_*α*_(*A*) is given by

14 $${S}_{\alpha }(A)={(1-\alpha )}^{-1}\sum _{l=1}^{M}\,\mathrm{log}(\,{\widehat{\nu }}_{l}^{\,\alpha }+{(1-{\widehat{\nu }}_{l})}^{\alpha }),$$

which can be interpreted as the dual of Eq. (6).

### The duality principle

As we have seen in the previous subsection, the Rényi entanglement entropy *S*_*α*_(*A*) can be computed in two equivalent ways, using the “coordinate” correlation matrix *C*_*A*_ and its “dual” ${\widehat{C}}_{A}$ (cf. Eqs (6–14)). This fact strongly suggests the existence of a deeper duality principle that applies to the reduced density matrix *ρ*_*A*_ itself, as evidenced by Eqs (5–13). To formulate this principle, we shall introduce the more precise notation *ρ*_*A*_(*K*) to denote the reduced density matrix of the subsystem *A* when the whole system is in the pure energy eigenstate |*K*〉 given by Eq. (2). It should be borne in mind that in this notation both sets *A* and *K* are subsets of {0, …, *N* − 1}, with the subindex always labelling the subsystem sites (in position space) and the argument the set of excited momenta. Let spec*T* stand for the spectrum of the matrix *T*, i.e., the set of its eigenvalues, each counted with its respective multiplicity. Likewise, we shall denote by spec_0_ *ρ* the spectrum of a density matrix *ρ* excluding its zero eigenvalues, i.e., ${{\rm{spec}}}_{0}\rho ={\rm{spec}}(\rho {|}_{{({\rm{\ker }}\rho )}^{\perp }})$. We shall then say that two density matrices *ρ*_*i*_ (*i* = 1, 2) *are similar up to zero eigenvalues* if spec_0_ *ρ*_1_ = spec_0_ *ρ*_2_, i.e., *ρ*_1_ and *ρ*_2_ have the same nonzero eigenvalues with the same multiplicities. We are now ready to state the following fundamental result:

**Theorem 1.** *The reduced density matrices ρ*_*A*_(*K*) *and ρ*_*K*_(*A*) *are similar up to zero eigenvalues*.

*Proof*. Indeed, by Eqs (5–13) the spectrum of *ρ*_*A*_(*K*) excluding the zero eigenvalues can be written in the two equivalent ways

15 $$\begin{array}{ccc}{{\rm{s}}{\rm{p}}{\rm{e}}{\rm{c}}}_{0}({\rho }_{A}(K)) & = & \{\prod _{l=1}^{L}{\nu }_{l}^{{\varepsilon }_{l}}{(1-{\nu }_{l})}^{1-{\varepsilon }_{l}}|{\varepsilon }_{l}\in \{0,1\},{\nu }_{l}\notin \{0,1\}\}\\  & = & \{\prod _{m=1}^{M}{\hat{\nu }}_{m}^{\,{\varepsilon }_{m}}{(1-{\hat{\nu }}_{m})}^{1-{\varepsilon }_{m}}|{\varepsilon }_{m}\in \{0,1\},{\hat{\nu }}_{m}\notin \{0,1\}\}.\end{array}$$

Let us denote by *C*_*A*_(*K*) and ${\widehat{C}}_{A}(K)$ the correlation matrix (4) and its dual version (9). We then have ${\widehat{C}}_{A}(K)={C}_{K}(A)$, ${C}_{A}(K)={\widehat{C}}_{K}(A)$, and consequently the sets ${\{{\nu }_{l}\}}_{l=1}^{L}$ and ${\{{\widehat{\nu }}_{m}\}}_{m=1}^{M}$ are interchanged by the duality transformation *A* ↔ *K*. Applying Eq. (15) to the reduced density matrix *ρ*_*K*_(*A*) we conclude that spec_0_(*ρ*_*A*_(*K*)) = spec_0_(*ρ*_*K*_(*A*)), as claimed.□

If *S* is any entropy functional, from now on we shall use the more precise notation *S*(*A*; *K*) = *S*(*ρ*_*A*_(*K*)). Obviously, from the Shannon–Khinchin axioms it follows that two density matrices which are similar up to zero eigenvalues necessarily have the same entropy. From this fact and the previous theorem one can immediately deduce the important *duality principle*

16 $$S(A;K)=S(K;A)\,,$$

valid for any entropy functional *S*.

As a first application of this general principle, we shall rigorously derive an asymptotic expression for the Rényi entanglement entropy of a subsystem *A* consisting of *r* > 1 disjoint blocks of consecutive spins when the set *K* of excited momenta is a single set of *M* consecutive integers, valid in the limit $N\gg M\gg 1$. More precisely, let $A={\cup }_{i=1}^{r}[{U}_{i},{V}_{i})$, *K* = [*P*, *Q*), where [*U*_*i*_, *V*_*i*_) denotes the set of all integers *l* such that ${U}_{i}\le l < {V}_{i}$ (so that the cardinal of [*U*_*i*_, *V*_*i*_) is *V*_*i*_ − *U*_*i*_), and similarly for [*P*, *Q*). We first let *N* → ∞ with *M* fixed and assume that the following limits exist:

$$\mathop{\mathrm{lim}}\limits_{N\to \infty }\frac{2\pi {U}_{i}}{N}\equiv {u}_{i},\quad \mathop{\mathrm{lim}}\limits_{N\to \infty }\frac{2\pi {V}_{i}}{N}\equiv {v}_{i},$$

with *u*_*i*_, *v*_*i*_ ∈ [0, 2*π*], *u*_*i*+1_ − *v*_*i*_ > 0, *v*_*r*_ − *u*_1_ < 2*π*. We shall be interested in the asymptotic behavior of the Rényi entropy *S*_*α*_ as *M* → ∞. Thus the problem at hand is precisely the dual of the one solved in refs 20, 24. with the help of the Fisher–Hartwig conjecture. One of the main results of the latter references can be recast in the present context as the asymptotic formula

17 $${S}_{\alpha }([U,V);{\cup }_{j=1}^{s}[{P}_{j},{Q}_{j}))\sim {b}_{\alpha }(s\,{\rm{l}}{\rm{o}}{\rm{g}}\,L+\sum _{j=1}^{s}\,{\rm{l}}{\rm{o}}{\rm{g}}(2\,\sin ({\textstyle \tfrac{{q}_{j}-{p}_{j}}{2}}))+\,{\rm{l}}{\rm{o}}{\rm{g}}\,f({\bf{p}},{\bf{q}}))+s{c}_{\alpha },$$

where $${p}_{j}\equiv \mathop{\mathrm{lim}}\limits_{N\to \infty }(2\pi {P}_{j}/N)$$, $${q}_{j}\equiv \mathop{\mathrm{lim}}\limits_{N\to \infty }(2\pi {Q}_{j}/N)$$,

18 $$\begin{array}{ccc}{b}_{\alpha } & = & \frac{1}{6}(1+\frac{1}{\alpha }),\quad {c}_{\alpha }=\frac{1}{1-\alpha }{\int }_{0}^{{\rm{\infty }}}(\alpha \,{{\rm{c}}{\rm{s}}{\rm{c}}{\rm{h}}}^{2}t-{\rm{c}}{\rm{s}}{\rm{c}}{\rm{h}}\,t\,{\rm{c}}{\rm{s}}{\rm{c}}{\rm{h}}(t/\alpha )-\frac{1-{\alpha }^{2}}{6\alpha }\,{e}^{-2t})\frac{dt}{t},\\  &  & f({\bf{p}},{\bf{q}})=\prod _{1\le i < j\le s}\frac{\sin ({\textstyle \tfrac{{q}_{j}-{p}_{i}}{2}})\sin ({\textstyle \tfrac{{p}_{j}-{q}_{i}}{2}})}{\sin ({\textstyle \tfrac{{p}_{j}-{p}_{i}}{2}})\sin ({\textstyle \tfrac{{q}_{j}-{q}_{i}}{2}})}\end{array}$$

and the ~ notation means that the difference between the LHS and the RHS tends to 0 as *L* → ∞. From the duality relation (16) and Eqs (17), (18) it then follows that when *M* → ∞ we have

19 $$\begin{array}{ccc}{S}_{\alpha }({\cup }_{i=1}^{r}[{U}_{i},{V}_{i});[P,Q)) & = & {S}_{\alpha }([P,Q);{\cup }_{i=1}^{r}[{U}_{i},{V}_{i}))\sim {b}_{\alpha }[r{\rm{l}}{\rm{o}}{\rm{g}}\,M\\  &  & +\sum _{i=1}^{r}\,{\rm{l}}{\rm{o}}{\rm{g}}(2\,\sin ({\textstyle \tfrac{{v}_{i}-{u}_{i}}{2}}))+\,{\rm{l}}{\rm{o}}{\rm{g}}\,f({\bf{u}},{\bf{v}})]+r{c}_{\alpha }.\end{array}$$

Taking into account that *f*(**u**, **v**) = 1 when *r* = 1, from the previous formula we obtain the remarkable relation

20 $${S}_{\alpha }({\cup }_{i=1}^{r}[{U}_{i},{V}_{i});[P,Q))\sim \sum _{i=1}^{r}{S}_{\alpha }([{U}_{i},{V}_{i});[P,Q))-{I}_{\alpha }({\bf{u}},{\bf{v}}),\,{\rm{w}}{\rm{i}}{\rm{t}}{\rm{h}}\,{I}_{\alpha }({\bf{u}},{\bf{v}})\equiv -{b}_{\alpha }{\rm{l}}{\rm{o}}{\rm{g}}\,f({\bf{u}},{\bf{v}})\,,$$

where the last term can be naturally interpreted as an asymptotic approximation to the *mutual information* shared by the blocks [*U*_1_, *V*_1_), …, [*U*_*r*_, *V*_*r*_). We believe that this is the first time that this asymptotic formula, which agrees with well-known CFT results, has been rigorously established using the (proved part of the) Fisher–Hartwig conjecture.

It is important to keep in mind the limiting process leading to Eq. (19) in order to correctly assess its limit of validity. For instance, using the connection between one-dimensional critical systems and 1 + 1 dimensional CFTs it follows that the asymptotic behavior of *S*_*α*_ is given (in our notation) by^11, 25^

21 $${S}_{\alpha }({\cup }_{i=1}^{r}[{U}_{i},{V}_{i});[P,Q))\sim {b}_{\alpha }[r\,{\rm{l}}{\rm{o}}{\rm{g}}(\frac{N}{\pi }\,\sin (\pi \,\frac{M}{N}))+\sum _{i=1}^{r}\,{\rm{l}}{\rm{o}}{\rm{g}}({v}_{i}-{u}_{i})+\,{\rm{l}}{\rm{o}}{\rm{g}}\,{f}^{({\rm{\infty }})}({\bf{u}},{\bf{v}})]+r{c}_{\alpha },$$

where *f* ^(∞)^(**u**, **v**) is the product of cross ratios

22 $${f}^{(\infty )}({\bf{u}},{\bf{v}})=\prod _{1\le i < j\le r}\frac{({v}_{j}-{u}_{i})({u}_{j}-{v}_{i})}{({u}_{j}-{u}_{i})({v}_{j}-{v}_{i})}.$$

The apparent discrepancy between the latter formulas and Eqs (18), (19) is easily explained taking into account that the limiting process in the latter references is the *dual* of the present one, namely *N* → ∞ with *fixed U*_*i*_, *V*_*i*_ and 2*πP*/*N* → *p*, 2*πQ*/*N* → *q*. In other words, Eqs (18), (19) apply when $N\gg M\gg 1$ and arbitrary *L* < *N*, while Eqs (21), (22) are valid for $N\gg L\gg 1$ and arbitrary *M* < *N*. It is also obvious that both approaches coincide in the (rather uninteresting) case in which both *M*/*N* and *L*/*N* tend to zero. On the other hand, it should be apparent that neither Eqs (18), (19) nor (21)-(22) are valid in the general situation in which *both L*/*N* and *M*/*N* tend to a nonzero limit as *N* → ∞. In fact, it is clear a priori that none of these formulas can hold in the latter range, since they are not consistent with the invariance under complements identity *S*(*A*; *K*) = *S*(*A* ^*c*^; *K*) and its dual consequence *S*(*A*; *K*) = *S*(*A*; *K* ^*c*^), where *A* ^*c*^ and *K* ^*c*^ respectively denote the complements of *A* and *K* with respect to the set {0, …, *N* − 1}.

**Figure 1:**
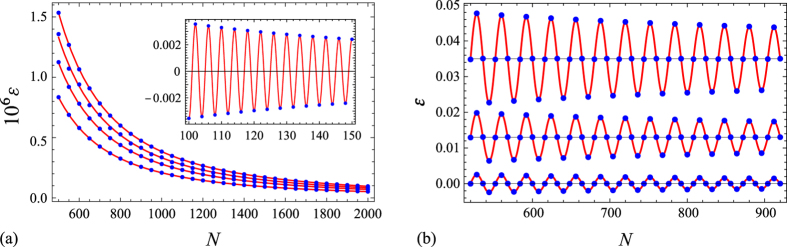
Difference *ε* between the exact value of the Rényi entropy, computed via Eq. (6) by numerical diagonalization of the correlation matrix (4), and its asymptotic approximation (23) for (**a**) *γ*_*x*_ = *γ*_*p*_ = 1/2 and (**b**) *γ*_*x*_ = 1/8, *γ*_*p*_ = 1/4. In panel (a) we have shown the cases (bottom to top) *α* = 3/5, 2/3, 3/4, 1 (von Neumann entropy) and *α* = 2 (inset), while panel (b) depicts the cases *α* = 2, 5/2, 3 (bottom to top, with the horizontal axis displaced respectively by 0.013 and 0.035 in the last two cases to avoid overlap). The solid red lines represent the curves providing the best fits of the data to the laws *aN* ^−2^ (main panel (a)) and *aN* ^−2/*α*^cos(2*πγ*_*x*_ *γ*_*p*_ *N*) (inset of panel (a) and panel (b)).

Our next objective is to find an extension of Eqs (19) and (21) valid in the general case in which both *L*/*N* and $M/N$ tend to nonzero limits *γ*_*x*_ and *γ*_*p*_ as *N* → ∞. To this end, consider first the simplest case in which *r* = *s* = 1. By translation invariance and criticality, as *N* → ∞ we must have ${S}_{\alpha }([U,V);[P,Q))\sim {b}_{\alpha }\,{\rm{l}}{\rm{o}}{\rm{g}}\,N+{\sigma }_{\alpha }({\gamma }_{x},{\gamma }_{p})$, where *γ*_*x*_ = (*V* − *U*)/*N*, *γ*_*p*_ = (*Q* − *P*)/*N* and *σ*_*α*_ satisfies: (i) *σ*_*α*_(*γ*_*x*_,*γ*_*p*_) = *σ*_*α*_(*γ*_*p*_,*γ*_*x*_) (on account of the duality principle (16)), (ii) *σ*_*α*_(*γ*_*x*_,*γ*_*p*_) = *σ*_*α*_(1 − *γ*_*x*_,*γ*_*p*_) (by the invariance of the entropy under complements), (iii) *σ*_*α*_(*γ*_*x*_,*γ*_*p*_) = *b*_*α*_log(2*γ*_*x*_sin(*πγ*_*p*_)) + *c*_*α*_ + *o*(1), with ${\mathrm{lim}}_{{\gamma }_{x}\to 0}o(1)=0$ (by Eq. (21) with *r* = 1). (In fact, combining conditions i) with ii) and iii) it immediately follows that *σ*_*α*_(*γ*_*x*_,*γ*_*p*_) = *σ*_*α*_(*γ*_*x*_, 1 − *γ*_*p*_) and *σ*_*α*_(*γ*_*x*_,*γ*_*p*_) = *b*_*α*_log(2*γ*_*p*_sin(*πγ*_*x*_)) + *c*_*α*_ + *o*(1), where *o*(1) → 0 as *γ*_*p*_ → 0.) Obviously, the simplest function satisfying the previous requirements is ${\sigma }_{\alpha }({\gamma }_{x},{\gamma }_{p})={b}_{\alpha }\,\mathrm{log}(\tfrac{2}{\pi }\,\sin (\pi {\gamma }_{x})\sin (\pi {\gamma }_{p}))+{c}_{\alpha }$, obtained from Eq. (21) with *r* = 1 by the replacement $\pi {\gamma }_{x}\mapsto \,\sin (\pi {\gamma }_{x})$. Numerical calculations show that for all *α* > 0 the correct asymptotic formula for *S*_*α*_([*U*; *V*); [*P*, *Q*)) is indeed the simplest one, namely

23 $${S}_{\alpha }([U,V);[P,Q))\sim {b}_{\alpha }\,{\rm{l}}{\rm{o}}{\rm{g}}(\frac{2N}{\pi }\,\sin (\pi {\gamma }_{x})\sin (\pi {\gamma }_{p}))+{c}_{\alpha }$$

(see, e.g., Fig. 1 (a) for the most “unfavourable” case *γ*_*x*_ = *γ*_*p*_ = 1/2). This conclusion is also in agreement with the analogous result in ref. 32 for the *XX* model. In fact, we found the leading correction to the approximation (23) to be monotonic in *N* and *O*(*N* ^−2^) for *α* = 1, and *O*(cos(2*πγ*_*x*_ *γ*_*p*_ *N*)*N* ^−2/*α*^) for *α* > 1 (cf. Fig. 1). This behaviour qualitatively agrees with the results of ref. 33 for the error of the Jin–Korepin asymptotic formula for the Rényi entanglement entropy of the ground state of the *infinite XX* chain (Eq. (23) with sin(*πγ*_*x*_) replaced by *πγ*_*x*_). On the other hand, in the case 0 < *α* < 1 (which was not addressed in the latter reference), our numerical calculations suggest that the correction to Eq. (23) is monotonic and *O*(*N* ^−2^).

At this point, it is very natural to assume that Eq. (20) and its dual are valid for *all* values of the parameters *γ*_*x*_,*γ*_*p*_ ∈ (0, 1), and not just for ${\gamma }_{p}\ll 1$ or ${\gamma }_{x}\ll 1$, respectively. The latter assumption and Eq. (23) thus lead to the asymptotic formulas

24 $${S}_{\alpha }({\cup }_{i=1}^{r}[{U}_{i},{V}_{i});[P,Q))\sim {b}_{\alpha }[r\,{\rm{l}}{\rm{o}}{\rm{g}}(\frac{2N}{\pi }\,\sin (\pi {\gamma }_{p}))+\sum _{i=1}^{r}\,{\rm{l}}{\rm{o}}{\rm{g}}\,\sin ({\textstyle \tfrac{{v}_{i}-{u}_{i}}{2}})]-{I}_{\alpha }({\bf{u}},{\bf{v}})+r{c}_{\alpha },$$

25 $${S}_{\alpha }([U,V);{\cup }_{i=1}^{s}[{P}_{i},{Q}_{i}))\sim {b}_{\alpha }[s\,{\rm{l}}{\rm{o}}{\rm{g}}(\frac{2N}{\pi }\,\sin (\pi {\gamma }_{x}))+\sum _{i=1}^{s}\,{\rm{l}}{\rm{o}}{\rm{g}}\,\sin ({\textstyle \tfrac{{q}_{i}-{p}_{i}}{2}})]-{I}_{\alpha }({\bf{p}},{\bf{q}})+s{c}_{\alpha }.$$

In fact, the validity of the latter equations can be justified by noting that one can go from Eq. (17), which holds for an *infinite* chain, to its analogue for a finite chain by the usual procedure^18, 32^ of replacing the “arc distance” *L* by the chord length (*N*/*π*)sin(*πL*/*N*) = (*N*/*π*)sin(*πγ*_*x*_). In this way Eq. (17) immediately yields Eq. (25), which implies its counterpart (24) by the duality principle (16).

**Figure 2:**
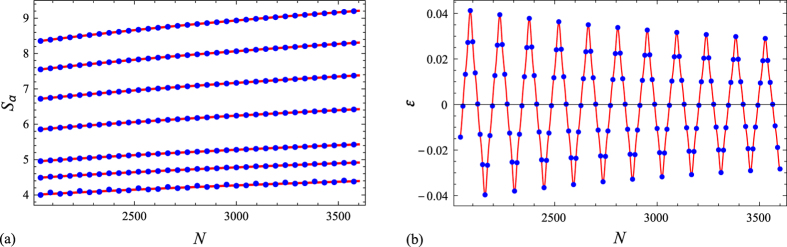
(**a**) Exact Rényi entropy *S*_*α*_ (blue dots) vs. its asymptotic approximation (24) (continuous red line) for a subsystem consisting of three equispaced blocks of equal length *N*/12 when the whole system’s state (2) is made up of a sequence of consecutive excited modes of length *N*/12 (*r* = 3, *s* = 1, *γ*_*x*_ = 1/4, *γ*_*p*_ = 1/12). The values of the Rényi parameter *α* considered are (from top to bottom) 1/2, 3/5, 3/4, 1, 3/2, 2 and 3. (**b**) Difference *ε* between the exact entropy *S*_3_ and its approximation (24) in the previous configuration as a function of the number of fermions *N*. The continuous red line is the graph of the function *f*(*N*)*N* ^−2/3^, with *f*(*N*) = −5.54238cos(*ν*_0_ *N*) − 0.742586cos(3*ν*_0_ *N*) − 0.39794cos(5*ν*_0_ *N*) and *ν*_0_ = 2*πγ*_*x*_ *γ*_*p*_/*r* = *π*/72.

Again, our numerical calculations for several block configurations and a wide range of values of the Rényi parameter *α* fully corroborate the validity of Eqs (24), (25) (see, e.g., Fig. 2). More precisely, our numerical analysis suggests that for sufficiently large *N* the error term in the latter equations behaves as *f*(*N*)*O*(*N* ^−min(2,2/*α*)^), where *f*(*N*) is a periodic function of *N*. In particular, the error term may not be monotonic in *N* even for $\alpha \le 1$, in contrast with what happens in the *r* = *s* = 1 case. The above results are in agreement with those reported in ref. 16 for the (infinite) *XY* chain and its corresponding free fermion model with *α* > 1, *r* = 2 and *s* = 1.

### Multi-block entanglement entropy: conjecture for the general case

We shall address in this section the general problem, in which both sets *A* and *K* consist of several blocks of consecutive sites or modes, respectively. To the best of our knowledge, an asymptotic formula for the entanglement entropy in this case has not previously appeared in the literature. As explained above, the main difficulty is now that neither the correlation matrix *C*_*A*_ nor its dual ${\widehat{C}}_{A}$ are Toeplitz, so that the standard procedure based on the use of the Fisher–Hartwig conjecture to obtain an asymptotic formula for the characteristic polynomial of the correlation matrix *C*_*A*_ (or of its dual ${\widehat{C}}_{A}$) is not applicable. Our approach for deriving a plausible conjecture for the asymptotic behavior of *S*_*α*_ in the general case considered in this subsection relies instead on the general duality principle discussed in the previous section (cf. Theorem 1 and Eq. (16)). In addition, we shall make the natural assumption that when the distance between any two consecutive blocks *A*_*i*_, *A*_*i*+1_ is much larger than the maximum block length (i.e., when ${\min }_{1\le i\le r}({u}_{i+1}-{v}_{i})\gg {\max }_{1\le i\le r}({v}_{i}-{u}_{i})$, where *u*_*r*+1_ ≡ *u*_1_ + 2*π*) the entanglement entropy is asymptotic to the sum of the single block entropies *S*_*α*_(*A*_*i*_; *K*). The motivation behind this assumption is that when the blocks are far apart their mutual influence should be negligible, and the Rényi entropy is of course additive over independent events.

The simplest asymptotic formula satisfying the above assumption is the trivial one ${S}_{\alpha }(A;K) \sim {\sum }_{i=1}^{r}{S}_{\alpha }({A}_{i};K)$. However, the latter formula cannot be correct, since it violates the duality principle. The obvious way of fixing this shortcoming would be to add the dual term ${\sum }_{j=1}^{s}{S}_{\alpha }(A;{K}_{j})$ to the RHS, but the resulting formula violates the above assumption. On the other hand, since by Eq. (20) ${\sum }_{j=1}^{s}{S}_{\alpha }(A;{K}_{j}) \sim {\sum }_{i=1}^{r}{\sum }_{j=1}^{s}{S}_{\alpha }({A}_{i};{K}_{j})-s{I}_{\alpha }({\bf{u}},{\bf{v}})$, and ${I}_{\alpha }({\bf{u}},{\bf{v}}) \sim 0$ when the blocks in coordinate space are far apart, the heuristic formula

26 $${S}_{\alpha }(A;K) \sim \sum _{i=1}^{r}{S}_{\alpha }({A}_{i};K)+\sum _{j=1}^{s}{S}_{\alpha }(A;{K}_{j})-\sum _{i=1}^{r}\sum _{j=1}^{s}{S}_{\alpha }({A}_{i};{K}_{j})$$

satisfies the above fundamental assumption. This relation is also clearly consistent with the duality principle (16), since the RHS of Eq. (26) is invariant under the exchange of the sets *A* and *K* on account of Theorem 1. We are thus led to conjecture that when *N* → ∞ the Rényi entropy of a configuration with *r* blocks *A*_*i*_ in coordinate and *s* blocks *K*_*j*_ in momentum space satisfies the previous relation. Using Eqs (20), its dual and Eq. (23) we immediately arrive at the closed asymptotic formula

27 $$\begin{array}{ccc}{S}_{\alpha }(A;K) & \sim  & rs({b}_{\alpha }\,{\rm{l}}{\rm{o}}{\rm{g}}(\frac{2N}{\pi })+{c}_{\alpha })+s({b}_{\alpha }\sum _{i=1}^{r}\,{\rm{l}}{\rm{o}}{\rm{g}}\,\sin ({\textstyle \tfrac{{v}_{i}-{u}_{i}}{2}})-{I}_{\alpha }({\bf{u}},{\bf{v}}))\\  &  & +r({b}_{\alpha }\sum _{i=1}^{s}{\rm{l}}{\rm{o}}{\rm{g}}\,\sin ({\textstyle \tfrac{{q}_{i}-{p}_{i}}{2}})-{I}_{\alpha }({\bf{p}},{\bf{q}})).\end{array}$$

**Figure 3:**
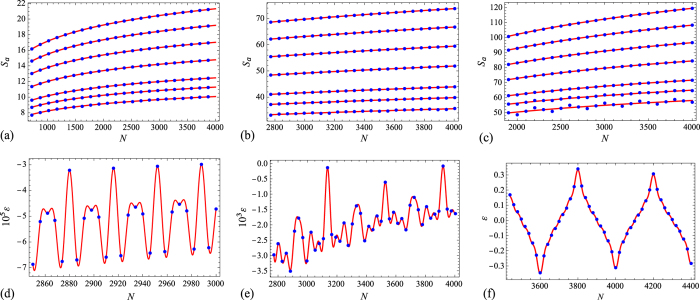
(**a–c**) Exact Rényi entropy *S*_*α*_ (blue dots) and its asymptotic approximation (27) (continuous red line) for *α* = 1/2, 3/5, 3/4, 1, 3/2, 2, 3 (top to bottom) in (**a**) a symmetric configuration with *r* = 3, *s* = 2, *γ*_*x*_ = 1/2, *γ*_*p*_ = 1/3, (**b**) an asymmetric configuration with *r* = 7, *s* = 4, *γ*_*x*_ = 1/2, *γ*_*p*_ = 1/4 (cf. Fig. 4), and (**c**) a symmetric configuration with *r* = 10, *s* = 5, *γ*_*x*_ = 1/2, *γ*_*p*_ = 1/4. (**d**–**f**) Difference *ε* between the exact entropy *S*_*α*_ and its approximation (27) for the above configurations and (**d**) *α* = 1/2, (**e**) *α* = 1 (von Neumann entropy), and (**f**) *α* = 2. The red lines represent the corresponding curves *f*(*N*)*N* ^−min(2,2/*α*)^, with $f(N)={\sum }_{k=0}^{{k}_{\max }}{a}_{k}\,\cos (k\nu N)$ given in Table 1.

**Figure 4:**
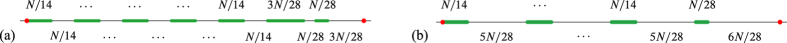
Asymmetric block configuration discussed in Fig. 3(b) in (**a**) coordinate space, (**b**) momentum space (the thick green lines represent the blocks, and the red dots are the two identified endpoints of the chain).

The latter equation is manifestly consistent with the duality principle stated in Theorem 1, as expected from the previous remark. It is also apparent that Eq. (27) reduces to Eq. (24) or (25) respectively for *s* = 1 or *r* = 1, as the asymptotic mutual information *I*_*α*_ vanishes for a single block. Moreover, it is straightforward to explicitly check that when the blocks in coordinate space are far apart the RHS reduces to the sum of the asymptotic approximations (25) to the single-block entropies *S*_*α*_(*A*_*i*_; *K*), since ${I}_{\alpha }({\bf{u}},{\bf{v}}) \sim 0$ in this limit. (By duality, a similar remark applies to the case in which the blocks [*P*_*j*_, *Q*_*j*_) in momentum space are far apart from each other.) Finally, it is immediate to check that Eq. (27) satisfies the invariance under complements identity. We have verified through extensive numerical calculations with a wide range of configurations in coordinate and momentum space that when $N\gg 1$ Eq. (27) is correct. In fact, for symmetric configurations (consisting of equally spaced blocks of the same length, both in coordinate and momentum space) the error term in the latter equation behaves as *f*(*N*)*N* ^−min(2,2/*α*)^, where *f* is again a periodic function. More precisely (for rational *γ*_*x*_ and *γ*_*p*_), *f*(*N*) is well approximated by a trigonometric polynomial ${\sum }_{k=0}^{{k}_{\max }}{a}_{k}\,\cos (k\nu N)$ with small *k*_max_ (independent of *N*), where the main frequency *ν* is the product of *ν*_0_ ≡ 2*πγ*_*x*_ *γ*_*p*_/*rs* with a simple fraction that can be computed from the configuration parameters *r*, *s*, *γ*_*x*_, *γ*_*p*_. The behavior of the error is very similar in non-symmetric configurations, except that in some cases it appears to decay faster than *N* ^−2^ for 0 < *α* < 1. As an example, in Fig. 3 we present our results for three different configurations with (*r*, *s*) = (3, 2), (7, 4), (10, 5). More precisely, the first and last of these configurations are symmetric, while the middle one is (slightly) asymmetric, as detailed in Fig. 4. As can be seen from Fig. 3(d–f), the error in Eq. (27) behaves in these three cases as described above, where the coefficients *a*_*k*_ of the trigonometric polynomial *f*(*N*) and its fundamental frequency *ν* are listed in Table 1.

**Table 1:** Coefficients *a*_*k*_ and fundamental frequency *ν* of the trigonometric polynomial $f(N)={\sum }_{k=0}^{{k}_{\max }}$ ${a}_{k}\,\cos (k\nu N)$ in the error of Eq. (27) for cases (d)-(f) in Fig. 3.

Case	*k*_max_	$({{\boldsymbol{a}}}_{{\bf{0}}}{\boldsymbol{,}}{\boldsymbol{\ldots }}{\boldsymbol{,}}{{\boldsymbol{a}}}_{{{\boldsymbol{k}}}_{{\bf{\max }}}})$	*ν*_0_	*ν*
(d)	2	(−438.485, 105.29, 66.716)	*π*/18	*ν*_0_
(e)	14	(−21790.1, 76.0009, 1602.85, 154.097, 5143.99, 397.121, 416.007, 1950.55, 4556.52, 156.444, 756.382, 168.572, 2164.74, 232.817, 2661.63)	*π*/112	2*ν*_0_/7
(f)	9	(0, −852.969, 0, −202.359, 0, −99.4396, 0, −57.2755, 0, −55.2294)	*π*/200	*ν*_*0*_

It should be noted that the asymptotic formula (27), which we have numerically checked for a *finite* chain, easily yields as a limiting case an analogous formula for an infinite chain. Indeed, if in Eq. (27) we let *γ*_*x*_ tend to 0 we have $\sin (({v}_{i}-{u}_{i})/2)\simeq \pi ({V}_{i}-{U}_{i})/N$, and similarly for the other arguments of the sine functions appearing in the asymptotic mutual information term *I*_*α*_(**u**, **v**). In this way we easily arrive at the analogue of Eq. (27) for an infinite chain, namely

28 $$\begin{array}{ccc}{S}_{\alpha }^{({\rm{\infty }})} & \sim  & s{b}_{\alpha }\,{\rm{l}}{\rm{o}}{\rm{g}}[\prod _{i=1}^{r}({V}_{i}-{U}_{i})\cdot \prod _{1\le i < j\le r}\frac{({V}_{j}-{U}_{i})({U}_{j}-{V}_{i})}{({U}_{j}-{U}_{i})({V}_{j}-{V}_{i})}]\\  &  & +r({b}_{\alpha }\sum _{i=1}^{s}\,{\rm{l}}{\rm{o}}{\rm{g}}\,\sin ({\textstyle \tfrac{{q}_{i}-{p}_{i}}{2}})-{I}_{\alpha }({\bf{p}},{\bf{q}}))+rs({b}_{\alpha }\,{\rm{l}}{\rm{o}}{\rm{g}}\,2+{c}_{\alpha }).\end{array}$$

To the best of our knowledge, this general asymptotic formula has not previously appeared in the literature. Note also that for *s* = 1 (i.e., when there is a single block of excited momenta) Eq. (28) implies the asymptotic expression for the mutual information of *r* blocks in coordinate space conjectured in ref. 25.

From the asymptotic approximation (27) (or its equivalent version Eq. (26)) one can also deduce a remarkable expression for the (asymptotic) mutual information of *r* blocks *A*_*i*_ ≡ [*U*_*i*_, *V*_*i*_) ($1\le i\le r$) in position space when the chain is in an energy eigenstate |*K*〉 made up of *s* blocks *K*_*j*_ ≡[*P*_*j*_, *Q*_*j*_) (1 ≤ *j* ≤ *s*) of excited momentum modes, defined as ${ {\mathcal I} }_{\alpha }({A}_{1},\ldots ,{A}_{r};K)\equiv {\sum }_{i=1}^{r}{S}_{\alpha }({A}_{i};K)-{S}_{\alpha }({\cup }_{i=1}^{r}{A}_{i};K)$. Indeed, using Eqs (20) and (26) we immediately obtain the asymptotic formula

29 $${{\mathscr{I}}}_{\alpha }({A}_{1},\ldots ,{A}_{r};K)\sim \sum _{j=1}^{s}\,[\sum _{i=1}^{r}{S}_{\alpha }({A}_{i};{K}_{j})-{S}_{\alpha }({\cup }_{i=1}^{r}{A}_{i};{K}_{j})]\sim \sum _{j=1}^{s}{I}_{\alpha }({\bf{u}},{\bf{v}})=s{I}_{\alpha }({\bf{u}},{\bf{v}}).$$

Thus (in the large *N* limit) the multi-block mutual information ${ {\mathcal I} }_{\alpha }$ is simply *s* times the mutual information when the chain’s state |*K*〉 consists of a single block of consecutive momenta. In particular, we see that ${ {\mathcal I} }_{\alpha }$ depends only on the *topo*log*y* of the state |*K*〉 (i.e., the number of blocks of excited momenta), not on its *geometry* (i.e., the particular arrangement and the lengths of these blocks). One could also define the mutual information of *s* blocks of excited momenta *K*_*j*_ ≡ [*P*_*j*_, *Q*_*j*_) ($1\le j\le s$) for a fixed configuration $A\equiv {\cup }_{i=1}^{r}{A}_{i}$ in position space. It easily follows from Eq. (29) and the duality principle that this mutual information is asymptotic to *rI*_*α*_(**p**, **q**). Of course, an analogous formula should hold for the infinite chain replacing the function *I*_*α*_ by its *N* → ∞ limit ${I}_{\alpha }^{(\infty )}({\bf{U}},{\bf{V}})={b}_{\alpha }\,\mathrm{log}\,{f}^{(\infty )}({\bf{U}},{\bf{V}})$. In particular, for *s* = 1 the latter expression implies that the model-dependent overall factor appearing in the general formula for the mutual information of a 1 + 1 dimensional CFT (see, e.g., refs 11, 13, 18) is equal to 1 for the models under consideration.

An alternative measure of the information shared by the blocks *A*_*i*_ ($1\le i\le r$) discussed in ref. 18 is the quantity ${\mathop{{\mathscr{I}}}\limits^{ \sim }}_{\alpha }({A}_{1},\ldots ,{A}_{r})\equiv {\sum }_{l=1}^{r}{(-1)}^{l+1}{\sum }_{1\le {i}_{1} < \ldots  < {i}_{l}\le r}{S}_{\alpha }({\cup }_{k=1}^{l}{A}_{{i}_{k}})$ (we omit the dependence on the chain’s state |*K*〉 for conciseness’s sake). In particular, for *r* = 3 we obtain the *tripartite information* introduced in ref. 12, whose vanishing characterizes the extensivity of the mutual information ${ {\mathcal I} }_{\alpha }$. It can be readily checked that the asymptotic relation (27) implies that ${\mathop{{\mathscr{I}}}\limits^{ \sim }}_{\alpha }({A}_{1},\ldots ,{A}_{r})$ vanishes asymptotically for the models under consideration. This follows immediately from Eq. (29) —which is itself a consequence of (27)— and the identities ${\sum }_{1\le {i}_{1} < \ldots  < {i}_{l}\le r}{\sum }_{k=1}^{l}{S}_{\alpha }({A}_{{i}_{k}})=(\genfrac{}{}{0ex}{}{r-1}{l-1}){\sum }_{i=1}^{r}{S}_{\alpha }({A}_{i})$, ${\sum }_{1\le {i}_{1} < \ldots  < {i}_{l}\le r}{I}_{\alpha }(({u}_{{i}_{1}},\ldots ,{u}_{{i}_{l}}),({v}_{{i}_{1}},\ldots ,{v}_{{i}_{l}}))=(\genfrac{}{}{0ex}{}{r-2}{l-2}){I}_{\alpha }({\bf{u}},{\bf{v}})$. In particular, this shows that the conjecture (27) implies the asymptotic extensivity of the mutual information ${ {\mathcal I} }_{\alpha }$ for the models under consideration. (For the infinite chain with *s* = 1, this had already been noted in ref. 25.)

Another noteworthy consequence of the asymptotic formula (27) is the fact that for large *N* the entanglement entropy can be approximately written as (omitting, for simplicity, its arguments)

30 $$\begin{array}{ccc}{S}_{\alpha } & \sim  & rs({b}_{\alpha }\,{\rm{l}}{\rm{o}}{\rm{g}}(\frac{2N}{\pi })+{c}_{\alpha })+{b}_{\alpha }g,\quad {\rm{w}}{\rm{i}}{\rm{t}}{\rm{h}}\quad g\equiv s(\sum _{i=1}^{r}\,{\rm{l}}{\rm{o}}{\rm{g}}\,\sin ({\textstyle \tfrac{{v}_{i}-{u}_{i}}{2}})+\,{\rm{l}}{\rm{o}}{\rm{g}}\,f(u,v))\\  &  & +r(\sum _{i=1}^{s}\,{\rm{l}}{\rm{o}}{\rm{g}}\,\sin ({\textstyle \tfrac{{q}_{i}-{p}_{i}}{2}})+\,{\rm{l}}{\rm{o}}{\rm{g}}\,f({\bf{p}},{\bf{q}})).\end{array}$$

The term in parenthesis in the latter formula, which contains the leading contribution *rsb*_*α*_log*N* to *S*_*α*_ as *N* → ∞, depends only on the topology of the configuration considered. In particular, from the coefficient of the log*N* term we deduce that the models under consideration are critical, behaving as a 1 + 1 dimensional CFT with central charge *rs*. Note also that the fact that the leading asymptotic behavior of the Rényi entanglement entropy *S*_*α*_ depends only on the topology of the configuration in *both* position and momentum space is a generalization of the widespread hypothesis (for the case *r* = 1) that the entanglement properties of critical fermion models are determined by the topology of their Fermi “surface” (see, e.g., ref. 34).

On the other hand, the numerical constant *g* in the previous equation is independent of *N* and *α*, and is solely determined by the geometry of the configuration in both position and momentum space. For instance, for the two symmetric configurations discussed in Fig. 3(a,c) this constant is respectively equal to −3log12 and −25log1250.

**Figure 5:**
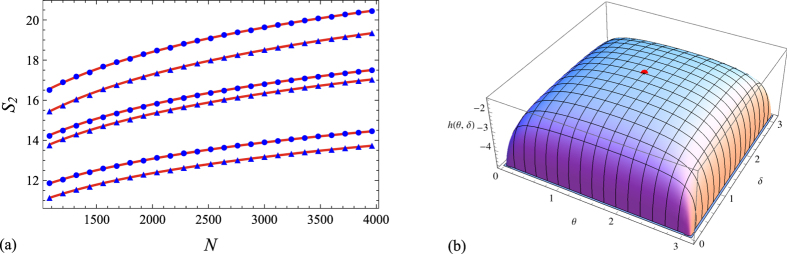
(**a**) Rényi entropy *S*_2_ vs. its asymptotic approximation (24) (red line) in symmetric (blue points) and some non-symmetric (blue triangles) configurations with *γ*_*x*_ = 1/3, *γ*_*p*_ = 1/2 and (bottom to top) 4 + 2, 5 + 2 and 4 + 3 blocks. (**b**) 3D plot of the function *h*(*θ*, *δ*) in Eq. (31) for *γ*_*x*_ = 1/2 (the red point corresponds to the symmetric configuration (*θ*, *δ*) = (*π*/2, *π*/2)).

The asymptotic formula (30) makes it possible to tackle several relevant problems that would otherwise be intractable in practice. For instance, it is natural to conjecture that fixing *r*, *s*, *γ*_*x*_ and *γ*_*p*_ the block configuration which maximizes the entropy is the symmetric one (i.e., *r* equally spaced blocks of equal length in position space, and similarly in momentum space). Our numeric calculations for several configurations suggest that this is indeed the case (see, e.g., Fig. 5(a) for the case *α* = 2). As we see from Eq. (30), this problem reduces to a standard (constrained) maximization problem for the geometric factor *g*, which in turns splits into two separate problems for the function ${g}_{1}({\bf{u}},{\bf{v}})\equiv {\sum }_{i=1}^{r}\,{\rm{l}}{\rm{o}}{\rm{g}}\,\sin ({\textstyle \tfrac{{v}_{i}-{u}_{i}}{2}})+\,{\rm{l}}{\rm{o}}{\rm{g}}\,f({\bf{u}},{\bf{v}})$ and its momentum space counterpart. For instance, when *r* = 2 we can express *g*_1_(**u**, **v**) in terms of the length *L*_1_ ≡ *V*_1_ − *U*_1_ of the first block and the interblock distance *d* ≡ *U*_2_ − *V*_1_ as

31 $${g}_{1}({\bf{u}},{\bf{v}})=\sigma (\theta )+\sigma (2\pi {\gamma }_{x}-\theta )+\sigma (2\pi {\gamma }_{x}+\delta )+\sigma (\delta )-\sigma (\theta +\delta )-\sigma (2\pi {\gamma }_{x}-\theta +\delta )\equiv h(\theta ,\delta ),$$

where *σ*(*x*) ≡ log sin(*x*/2), *θ* = 2*πL*_1_/*N* ∈ (0, 2*πγ*_*x*_), *δ* = 2*πd*/*N* ∈ (0, 2*π*(1 − *γ*_*x*_)). Moreover, from the symmetry of *h* under $\theta \mapsto 2\pi {\gamma }_{x}-\theta$ and $\delta \mapsto 2\pi (1-{\gamma }_{x})-\delta$, it suffices to find the maximum of this function in the rectangle (0, *πγ*_*x*_) × (0, *π*(1 − *γ*_*x*_)). An elementary calculation shows that *h* has a local maximum at *θ* = *πγ*_*x*_, *δ* = *π*(1 − *γ*_*x*_), i.e., at the symmetric configuration, and that ∇*h* has no other zeros on (0, *πγ*_*x*_] × (0, *π*(1 − *γ*_*x*_)]. This proves the conjecture in the case *r* = 2 (cf. Fig. 5(b)). For instance, for *r* = *s* = 2 the maximum value of the entropy is easily found from the latter argument and Eq. (30) to be 4[*b*_*α*_log(*N*sin(*πγ*_*x*_)sin(*πγ*_*p*_)/2*π*) + *c*_*α*_].

## Discussion

In this work we have rigorously formulated a general duality principle which posits the invariance of the Rényi entanglement entropy *S*(*A*; *K*) of a chain of free fermions under exchange of the sets of excited momentum modes *K* and chain sites *A* of the subsystem under study, where both *A* and *K* are the union of an arbitrary (finite) number of blocks of consecutive sites or modes. By means of this principle, we have derived an asymptotic formula for the Rényi entanglement entropy when the set *K* consists of a single block. From this formula and a natural assumption concerning the additivity of the entropy when the blocks are far apart from each other in either position or momentum space we have conjectured an asymptotic approximation for the entanglement entropy in the general case when both sets *A* and *K* consist of an arbitrary number of blocks. We have presented ample numerical evidence of the validity of this formula for different multi-block configurations, and have analyzed its error comparing it with its counterpart for the *XX* model discussed by Calabrese and Essler^33^. Our conjecture also yields an asymptotic formula for the mutual information of a certain number of blocks in position (or momentum) space valid for arbitrary multi-block configurations, which for *s* = 1 and in the case of an infinite chain is consistent with the general one for 1 + 1 dimensional CFTs.

The previous results open up several natural research avenues. In the first place, it would be desirable to find a rigorous proof of the fundamental asymptotic relation (26), which leads to the explicit asymptotic formula (27). In particular, it would be of interest to determine the range of models for which this relation holds. Another related problem is to study analytically the precise behavior of the error term in the latter equation. Indeed, our numerical results suggest that this error exhibits a qualitatively similar but considerably more complex behavior than its analogue for an infinite chain with a single block in both position and momentum spaces studied in ref. 33. Finally, an interesting question arising from the discussion after Eq. (30) is the analysis of the configurations *minimizing* the entropy with appropriate constraints, which could be naturally regarded as akin to “semiclassical” states.

### Note added in proof

After this article was submitted for review, the authors became aware of the paper by C.H. Lee, P. Ye and X.-L. Qi (J. Stat. Mech.-Theory E. (2014) P10023), in which an alternative proof of Theorem 1 based on previous results of Z. Huang and D.P. Arovas (Phys. Rev. B 86 (2012) 245109) is presented.
